# Evaluation of Commercial Corrosion Sensors for Real-Time Monitoring of Pipe Wall Thickness under Various Operational Conditions

**DOI:** 10.3390/s22197562

**Published:** 2022-10-06

**Authors:** Dong-Ho Shin, Hyun-Kyu Hwang, Heon-Hui Kim, Jung-Hyung Lee

**Affiliations:** 1 Graduate school, Mokpo National Maritime University, Mokpo 58628, Korea; 2 Division of Marine Engineering, Mokpo National Maritime University, Mokpo 58628, Korea

**Keywords:** corrosion, wall-thinning, ER and LPR sensor, UT sensor, electrochemical experiment

## Abstract

In this study, we investigated the performance and reliability of commercial corrosion sensors for monitoring the integrity of piping systems in various fluid environments as an alternative to ultrasonic transducers. To this end, we investigated pipes’ wall-thinning using commercial electrical resistance (ER), linear polarization resistance (LPR), and ultrasonic transducer (UT) sensors under various operating environments. A pilot-scale closed-loop test bed was built to simulate a real pipeline flow situation, from which the sensor data were collected and analyzed. Experimental results indicate that, in the case of the LPR sensor, it is challenging to accurately measure the corrosion rate when a specific measure exceeds the threshold in a severe corrosion environment. In contrast, the ER sensor could measure metal loss under all conditions and reflect the corresponding characteristics. The metal loss (about 0.25 mm) of the real pipe after the experiment was confirmed to be equal to the metal loss (0.254 mm) measured by the sensor. Furthermore, the regression analysis revealed a high correlation between the results obtained from the ER and UT sensors. Thus, evaluating the remaining thickness of the piping system using the commercial ER sensor is deemed to be effective and reliable.

## 1. Introduction

Piping systems play a vital role in many industries as a transport medium. In utility industries, piping is used for the water supply, tanks, heating and cooling water, and gas transfer. In chemical industries, especially in petroleum facilities, piping is applied to connect raw or refined chemicals to all components, such as the tanks and distillation facilities associated with each plant [1]. During the operation of the piping system, risks involve wall-thinning in the pipeline, which can lead to the leakage of hazardous chemicals induced by a puncture or rupture in the piping system [2].

Leakage occurs in these piping systems for various reasons. The American Water Association (AWWA) classified the causes of leaks as poor or defective materials, excessive pressure, errors in valve operation (opening and closing), leaks caused by corrosion and erosion, and accidental or intentional damage, which are considered to be continuous, gradual, or unexpected [3]. Among the various causes, most pipe leakages occur due to wall-thinning attributed to chemical and physical deterioration. The reason for this wall-thinning is corrosion and erosion, which causes unexpected leakage due to the continuous decrease in the thickness of pipe materials [3]. Corrosion and erosion reactions in pipes appear differently depending on the pipe’s intended use. The corrosion is the reaction between a material and surrounding environment, and thus can be accelerated depending on the pH, operating temperature, and the presence or absence of chloride and sulfate ions. Generally, the pipe damage caused by corrosion appears to be larger and faster than other damage types [4].

To date, most leak accidents have obtained attention from pipeline users or the mass media, and there are often no programs to monitor and manage such accidents [5]. This habituated negligence may lead to accidents due to pipe leaks, which have continued to occur. In 2008, at the Kori Nuclear Power Plant Unit 3, which was in regular operation in Korea, a leak occurred at the welding part of the drain valve on the B side of the steam generator. The staff responded to the accident by manually stopping it [6,7].

Water distribution networks also possess a risk of leakage. The leak rate was reported as 10.8% (about 720 million m^3^) of the total transported water nationwide. This leakage could account for approximately USD 658 million of the annual production cost [8]. As stated above, taking measures after a leak accident induces financial losses, environmental issues, and human costs. To prevent such accidents, it is essential to have prognostics and health management capabilities for integrity evaluations through condition-based monitoring of the piping system. 

Piping systems exhibit different pipe wall-thinning behaviors according to their various uses and environments. In particular, corrosion behavior by the components contained in the fluid appears to depend on industrial applications. Numerous studies have explored the factors affecting pipe wall-thinning and sensor technology to monitor the condition of the piping system and evaluate of the integrity of the piping system.

Diao et al. used four factors (salinity, pH, dissolved oxygen, and temperature) that affect the corrosion and chemical composition of alloys as input data to develop a corrosion rate (CR) prediction model for low alloys. The correlation between the input data and CR was investigated based on machine learning algorithms and feature pre-processing methods. Consequently, the multiple correlation coefficient (r^2^) of the actual CR value and the predicted value was 0.9 or higher. They reported that this could be an excellent tool for developing a CR prediction model algorithm through machine learning [9]. Sayed studied the internal corrosion of piping systems as function of flow rate and reported that this is one factor accelerating corrosion [10]. He also associated the failure mechanism of the piping system with flow-enhanced corrosion.

Different types of sensors have been developed to directly evaluate piping system integrity. Among the sensors employed to monitor pipe integrity, corrosion sensors, acoustic emission, and ultrasonic transducer (UT) sensors exhibit moderate performances when monitoring the corrosion and wall-thinning of pipes [11]. The UT sensor employs an operator to measure the remaining thickness of a pipe at a certain period in the field [12,13]. Measurements of the remaining pipe thickness using the UT sensor enable the determination of its remaining useful life. The sensor has some disadvantages, including a limited measurement range according to the size of the sensor, and poor repeatability of measurements depending on the skill level of the operator [14,15]. More importantly, measurements cannot be achieved when the operator cannot access the target in real-time, such as in a buried pipe [16], and a measurement error may be produced by the UT sensor when there is fluid flow in the pipe [17].

Unlike the UT sensor, the corrosion sensor measures the CR of the surrounding environment, which enables the lifetime of the pipe to be determined. This does not require a skilled operator [18] and is the most widely used sensor for predicting the pipeline conditions. The sensor converts the electric signal using the transmitter [19]. Representative corrosion sensors include electrical resistance (ER) and linear polarization resistance (LPR) sensors. The ER sensor generates a signal corresponding to the metal loss; the CR can be obtained by calculating the slope of the damage curve [18]. Compared to other sensors, a conventional corrosion sensor can be applied to a relatively high-temperature environment, which can be used to evaluate the effect of erosion [20,21]. The LPR sensor has widespread use. It is convenient for the interpretation of data, as it directly calculates and provides CR for the environment [18]. A growing number of the literature has investigated ER and LPR sensors as techniques to monitor internal corrosion rate.

Joosten et al. proposed a dual-element ER sensor for high-sensitivity corrosion monitoring technology and evaluated its stability in various environments. In their study, the sensor showed an excellent performance in measuring the CR, and the influence of the temperature was discussed [22]. Durrani et al. used a corrosion coupon and LPR sensor to calculate the CR of a piping system. They claimed that, with an LPR sensor equipped with a corroded electrode, over-predicted CR values from 20 to 44% could be induced [23], indicating that measurement errors can be produced while the sensor is in use.

Previous studies have explored the feasibility of prototype ER or LPR sensors in limited and controlled environments. Although several commercial sensors are available on the market, few empirical studies have addressed their application to real-world operating environments. To ensure the commercial viability of the corrosion sensors, the reliability and measurement accuracy must be verified under different operating conditions in various fluid environments. The proper selection of a sensor for pipeline integrity monitoring requires consideration of the cost, availability, physical and chemical properties of the fluid, and human resources for maintenance.

In this study, we investigated the performance and reliability of commercial corrosion sensors for monitoring the integrity of the piping system in various fluid environments as an alternative to the UT sensor. The electrochemical evaluation was conducted to compare the measurement accuracy of the ER and LPR sensors compared to the UT sensor. A comparative analysis of the data obtained from these sensors was performed to verify the applicability of the corrosion sensors to monitoring wall thickness changes in pipes. Furthermore, statistical methods, such as the regression analysis and Pearson correlation, were employed to examine the influence of operating parameters on the sensor response. The findings of this study suggest further development directions for corrosion sensors, and is a useful reference for their industrial application.

## 2. Principle of Corrosion Sensor

### 2.1. Sensors for Piping System Condition and Wall-Thinning Monitoring

#### 2.1.1. ER Sensor

**Figure 1:**
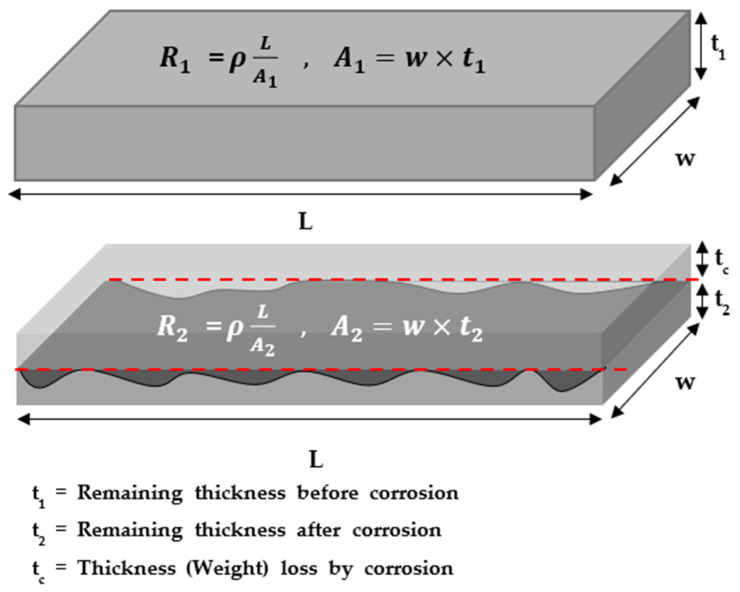
Sensing principle of electric resistance (ER) sensor by thickness reduction.

The ER sensor measures an electric resistance value and transmits an output signal as a damaged thickness value. This is similar to measuring the CR with the weight loss method [24]. The CR measurement using the weight loss method is performed by exposing the coupon to the actual corrosive environment or similar environments. This method measures the damage by assessing the coupon’s weight loss after the experiment. Similarly, the principle of the ER method is that when the sensor’s thickness is damaged by corrosion or erosion, the shape of the sensor changes, and the measured ER increases [25]. The change in the sensor resistance is expressed in the following equation, which is illustrated in Figure 1 [26]. The resistance of the fresh sensing element (R_1_) before thickness reduction is represented by t_1_. As corrosion progresses, the thickness of the element is reduced from t_1_, which is the initial thickness, to t_2_. Eventually, the thickness reduction (t_c_) leads to an increase in the resistance from R_1_ to R_2_, as shown in the equation in Figure 1. The change in the resistance of the ER sensor corresponds to a change in the metal loss.

**Figure 2:**
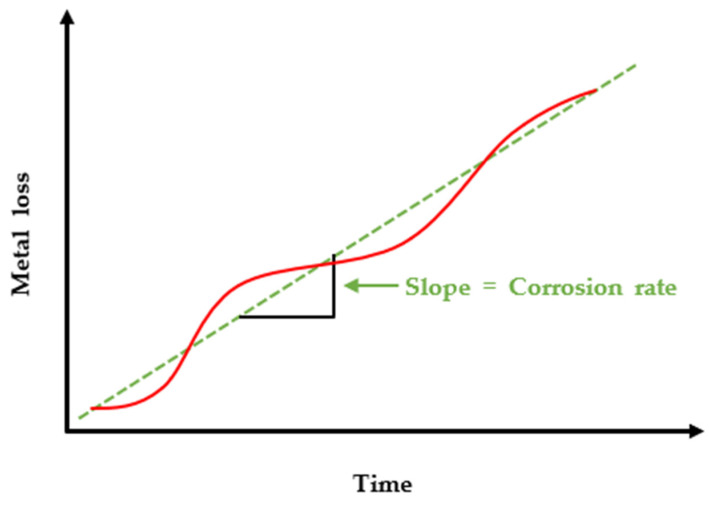
An example of metal loss versus time curve and a slope indicating corrosion rate.

The advantage of the ER method lies in the possibility of monitoring the damaged thickness of the pipe in real-time by measuring the resistance value that changes over time based on the initial resistance value. As shown in Figure 2, continuous measurements of metal loss generate curves, where the slope gives the value of CR. Since metal losses under the same environments may produce different CR over different periods of time, overall CR can be determined by a linear fitting of the datapoints on the graph.

**Figure 3:**
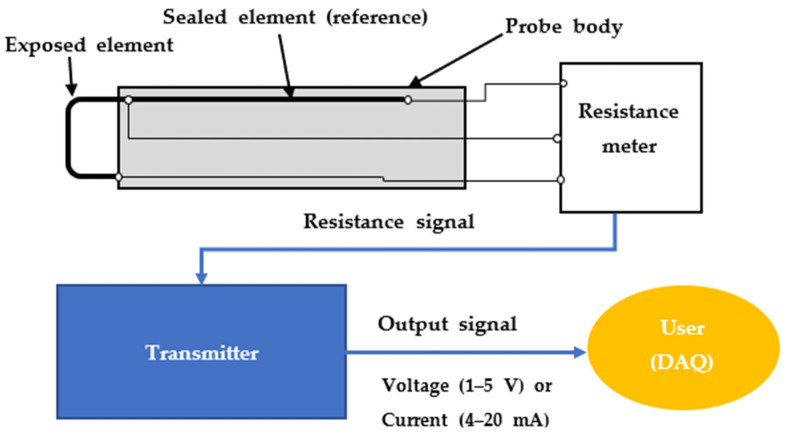
Schematic diagram of ER sensor and data acquisition system.

Figure 3 illustrates the schematic diagram of a typical ER sensor [27]. The general operating method of the ER sensor involves transmitting a constant current to the exposed and reference elements. Then, the resistance meter transmits the output value of the specific resistance from the exposed element. If the exposed element is damaged by the environment in which the ER sensor is placed, it transmits an increased output resistance. In this way, the resistance output of the exposed element is measured by a resistance meter according to the thickness of the damaged element. The measured resistance calculates the resistance corresponding to the damaged thickness through the dedicated transmitter and transmits it as a voltage (1–5 V) or current (4–20 mA) signal to users. Users continuously acquire the damaged thickness data of the ER sensor with the signal transmitted using a data acquisition (DAQ) system. Thus, a graph can be drawn and used to calculate the CR. Nevertheless, acquiring damaged thickness data over a certain period is necessary to estimate the CR using the ER sensor. There is a disadvantage in secondary data processing being required after the experiment and data acquisition. Further, the thickness of the ER sensor element is proportional to its service life. Therefore, users must employ a thick ER sensor element for long-term use. However, some reports claimed that the sensitivity of the sensor may be degraded when a thick element is used [20]. According to Ref. [20], if the element thickness of the sensor is manufactured in a thin form, it can be applied to an environment with a low CR. Li et al. reported that the highly sensitive thin film electric resistance sensor, developed in the form of a thin film, showed a good performance when applied to various corrosive environments [28]. Therefore, the user must accurately identify the environment in which the sensor is to be used and select the element thickness of the sensor accordingly.

#### 2.1.2. LPR Sensor

The LPR sensor employs the principle of the linear polarization resistance method [29]. The LPR method can simply and quickly calculate a wide range of CRs [30]. Thus, it is used in various fields owing to the high accuracy of the measured values. As described above, with the LPR method, the CR can be quickly calculated and monitored such that the user can immediately respond to emergencies [31]. Further, because the LPR method is based on an electrochemical process, it is generally performed with three electrodes (the reference, counter, and working electrode), and uses the correlation between the current density and the potential generated by the electrochemical reaction between the electrodes.

The LPR sensor works by applying a small voltage and measuring the ratio of voltage to current. When a power source is applied to the sensor electrodes, the current flows from the working electrode to the counter electrode in electrolyte solution. To determine polarization resistance, the current density and potential should first be determined. Therefore, the current density of the working electrode opposite the counter electrode is measured, and, at the same time, the potential of the working electrode is measured against the reference electrode. The measured current density value is inversely proportional to the ER value according to Ohm’s law.

**Figure 4:**
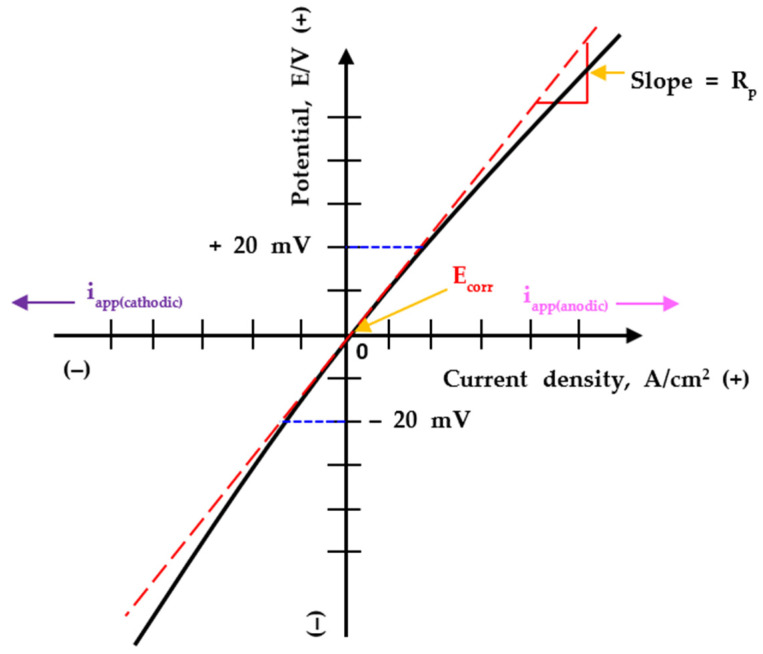
Hypothetical linear polarization resistance curve and polarization resistance (*R_p_*).

For example, assume that a low potentiodynamic polarization is applied between the working and reference electrodes. Then, the current flows from the working electrode to the counter electrode in the electrolyte solution. If the current density between the working and counter electrodes is high, the electrical resistance of the working electrode is said to be low. This means that the working electrode is easily ionized and the CR is fast [27]. Using this principle, the LPR sensor calculates the polarization resistance (*R_p_*) from the linear slope of the relationship between the overpotential and current density by using the overpotential within ± 20 mV at the maximum from the corrosion potential (*E_corr_*) of the working electrode, measured against the reference electrode [27,32]. Furthermore, by using the calculated *R_p_*, the corrosion current density (*i_corr_*) and CR are calculated in real-time, and CR data can be acquired through a transmitter dedicated to the LPR sensor. Consequently, the LPR sensor can only measure the CR when the electrochemical reaction (relationship between overvoltage and current) between the metal and aqueous solution has a linear relationship (Tafel behavior) [33]. Figure 4 shows a typical potential-current density graph for metallic materials in a corrosive environment [34]. The relationship between the potential-current density is linear near the *E_corr_*, which yields the *i_corr_*. This can be derived according to the Stern–Geary equation and uses the mixed potential theory [27,34]. The relationship between the current density and overpotential in a solution following the Tafel behavior is shown in the graph [35].

(1) $$E_{a}=\beta_{a}\left(\log\frac{i_{a}}{i_{corr}}\right)$$

(2) $$E_{c}=\beta_{c}\left(\log\frac{i_{c}}{i_{corr}}\right)$$

(3) $$E_{a}=E_{a}-E_{corr},\;E_{c}=E_{c}-E_{corr}$$

$\beta_{a}$: Tafel constant of anodic reaction (slope of straight line in E–logI curve).
$\beta_{c}$: Tafel constant of cathodic reaction (slope of straight line in E–logI curve).
$E_{a}$: Tafel constant of anodic reaction (slope of straight line in E–logI curve).
$E_{c}$: Tafel constant of cathodic reaction (slope of straight line in E–logI curve).

(4) $$i_{app.\;c}=i_{c}-i_{a}\left(\log\frac{i_{c}}{i_{corr}}\right)$$

$i_{app.\;c}$: Applied cathodic current density.
$i_{a}$: Current density of anodic oxidation reaction.
$i_{c}$: Current density of cathodic reduction reaction.

If Equations (1) and (2) are fixed in exponential form and then substituted into Equation (4), Equation (5) is obtained.

(5) $$i_{app.\;c}=i_{corr}\left(10^{-\frac{\varepsilon_{c}}{\beta_{c}}}-10^{\frac{\varepsilon_{a}}{\beta_{a}}}\right)$$

$\varepsilon_{a}$: Anodic overpotential.
$\varepsilon_{c}$: Cathodic overpotential.

Equation (5) approaches a straight line as the overpotential becomes close to zero (*E_corr_*). Hence, when it is differentiated, the equation for *R_p_* is obtained as follows.

(6) $$R_{p}={\left[\frac{d_{\varepsilon }}{di_{app}}\right]}_{\varepsilon \to 0}-{\left[\frac{\Delta_{\varepsilon }}{\Delta i_{app}}\right]}_{\varepsilon \to 0}=\frac{\beta_{a}\beta_{c}}{2.3\;i_{corr}\left(\beta_{a}+\beta_{c}\right)}=\frac{B}{i_{corr}}$$

(7) $$B=\frac{\beta_{a}\beta_{c}}{2.3\left(\beta_{a}+\beta_{c}\right)}$$

*B* = Tafel proportionality constant.

The CR is calculated according to the Faraday’s law of the *i_corr_*, calculated through Equation (5), and Equation (8) is used to calculate the CR is as follows.

(8) $$\mathrm{CR}=K_{1}\frac{i_{\mathrm{corr}}}{\rho }\mathrm{EW}$$

$K_{1}=3.27\times 10^{-3},\;\mathrm{mm}\cdot g/\mu A\cdot \mathrm{cm}\cdot \mathrm{yr}$
$i_{\mathrm{corr}}=\mathrm{Corrosion}\;\mathrm{current}\;\mathrm{density}\;$
$\rho =\mathrm{Density}\;\mathrm{in}\;g/{\mathrm{cm}}^{3}$
EW=Equivalent weight

The advantage of the LPR sensor is that, compared to the ER sensor, the CR can be directly monitored without additional data-processing. However, a disadvantage is that it may be difficult to accurately measure the CR when the environment in which the electrode is placed does not form a linear relationship between overpotential and current density [34,36]. Hence, the CR measurement using the LPR sensor can only be accurately measured when the electrochemical reaction of the electrode follows a constant reaction rate mechanism (Tafel behavior). Further, although the performance may differ depending on the maker, the LPR sensor has limitations in severely corrosive environments, as it can generally measure up to a maximum CR of 200 mils penetration per year (MPY).

**Figure 5:**
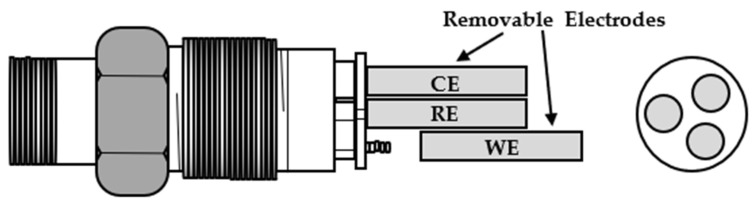
Schematic diagram of LPR sensor with three removable electrodes (CE, RE and WE).

Figure 5 illustrates a schematic diagram of the three electrode probes type of a com-mercial LPR sensor [27]. The probe of the LPR sensor consists of three electrodes (working, counter, and reference electrodes) made of the same metal. They can easily be assembled or disassembled using screws [27].

## 3. Experimental Methods

### 3.1. Test Bed for Simulating Piping System

**Figure 6:**
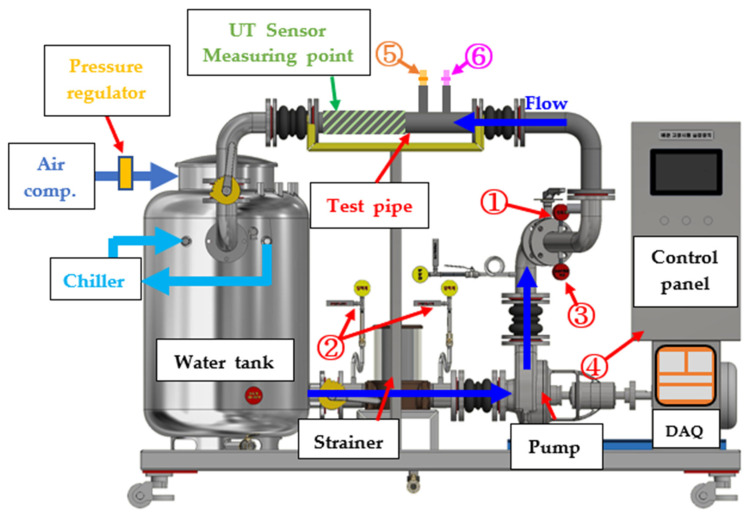
Schematic diagram of test bed for evaluating commercial corrosion sensors under various operational conditions: 1: pressure sensor, 2: D.P sensor, 3: temperature sensor, 4: motor current sensor, 5: ER sensor, 6: LPR sensor.

A test bed was designed and constructed to monitor wall-thinning damage to the pipe by corrosion, as shown in Figure 6. The test bed consisted of a water tank, strainer, centrifugal pump, valves, and metal piping to simulate the piping system. An air compressor was connected to the water tank to ensure the smooth operation of the centrifugal pump, and constant pressure was maintained using a pressure regulator. As the movement of the fluid generates frictional heat from the centrifugal pump, causing the fluid temperature to increase, the temperature of the fluid in the tank was kept constant using an external chiller. To measure the degree of corrosion damage, the grade SS275 (carbon steel) straight pipe was used as the test pipe for thickness measurements and analysis, and grade 304 stainless steel was used for the rest of the piping system. The carbon steel was chosen as the test material because it is widely used as a pipeline material in various industries due to its high strength, durability, moderate corrosion resistance, and cost-effectiveness [37,38,39].

The test bed was equipped with various sensors, including a pressure sensor (1), differential pressure sensor (2), temperature sensor (3), pump motor current sensor (4), ER sensor (5), and LPR sensor (6) to monitor the operating conditions and corrosion degree of the piping system. Table 1 lists the measurement ranges and output signals of these sensors.

The pipeline had specially designed ports for the flush mounting of the corrosion sensors. The corrosion sensors that were used are commercially available and their sensing element was made of carbon steel so that the sensor’s response could be correlated with the wall thinning of the carbon steel test pipe. Corrosion sensors (ER and LPR) can measure the metal loss, and thus determine CR in real-time. As shown in Table 1, the ER sensor can measure the metal loss in the range of 0–10 mils, and the LPR sensor was set to measure the CR of 0–200 MPY.

Generally, as the corrosion reaction of the piping system occurs, or the chloride concentration increases, metals or chloride are dissolved in the test solution, resulting in a decrease in pH and an increase in electrical conductivity. To measure such changes, pH and electrical conductivity were measured after sampling the test solution in a tank under changing test conditions. The measured data were interpolated and compared with ER and LPR sensor data.

### 3.2. Data Acquisition from Sensors

**Figure 7:**
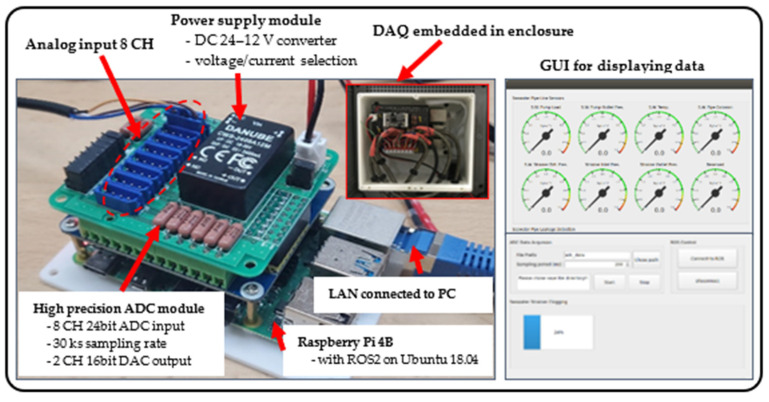
Photo of the DAQ system and the GUI for monitoring and control of sensor data.

The DAQ system was developed using a low-cost embedded system (Raspberry Pi 4B) interfaced with an analog-digital converter (resolution: 24 bit, sample rate: 30 Ks) to process the sensor signals (Figure 7). The system used a power supply module (24 VDC) for sensors with a 4–20 mA current loop or a 0–5 V signal. Linux (Ubuntu 18.04) was chosen as the operating system for the unit, and robot operating system (ROS2, Foxy) was chosen as the middleware framework. The ROS was used as a platform for data acquisition as it provides various functionalities and interfaces. In an ROS network, each sensor has its own node, which is the basic unit of execution in ROS. The ROS nodes establish the network by sending and receiving messages on a topic using a publish–subscribe communication model. The real-time sensor data were transmitted to the PC via an LAN for monitoring and then stored on the PC. The graphic user interface was built to display real-time data and operation using PyQT5, one of the cross-platform interface toolkit. All components for the DAQ system were packaged in a three-dimensional (3D)-printed enclosure designed for wall mounting near the test bed.

### 3.3. Test Profile for Pipe Condition Monitoring

**Figure 8:**
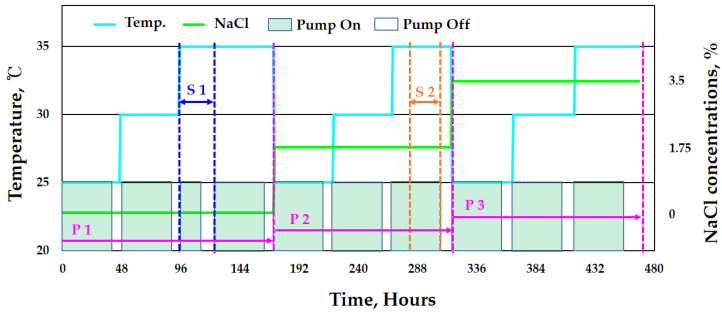
Proposed test profile with three programs including two failure scenarios (P1: NaCl 0%, P2: NaCl 1.75%, P3: NaCl 3.5%, S1: failure of piping system, S2: failure of DAQ communication).

Figure 8 illustrates the test profile of this study. To monitor the conditions and acquire data in various environments of the actual piping system, three test sets were applied in sequential order. Further, two failure scenarios were selected to realize the failure of the piping system and the DAQ system that may occur during operation in the field, and the piping system data were acquired simultaneously.

To analyze the environmental factors affecting the wall-thinning of the piping system, the NaCl concentration, pump start/stop, and fluid temperature were chosen as variables. In the first test set (P1), the start/stop of the pump was repeated in the order of fluid temperature 25, 30, and 35 °C at a chloride concentration of 0%. This scenario was used to acquire the piping system data in a situation where the piping is empty due to failure or maintenance. In the first test set scenario (S1), the data were acquired from various sensors after an emergency stop of the pump under 0% chloride concentration and 35 °C fluid temperature. In the second test set (P2), the experiment was performed under the same conditions as in the first test set at a chloride concentration of 1.75%. In the second scenario (S2), the connection cable between the sensor and the data acquisition device was intentionally disconnected at a chloride concentration of 1.75% and a fluid temperature of 35 °C to acquire status data according to the failure or poor connection of the sensor, DAQ device, and connection cable. In the third test set (P3), the test was performed with 3.5% chloride concentration, all other conditions being the same.

### 3.4. UT Sensor for Measuring Test Pipe Thickness 

**Figure 9:**
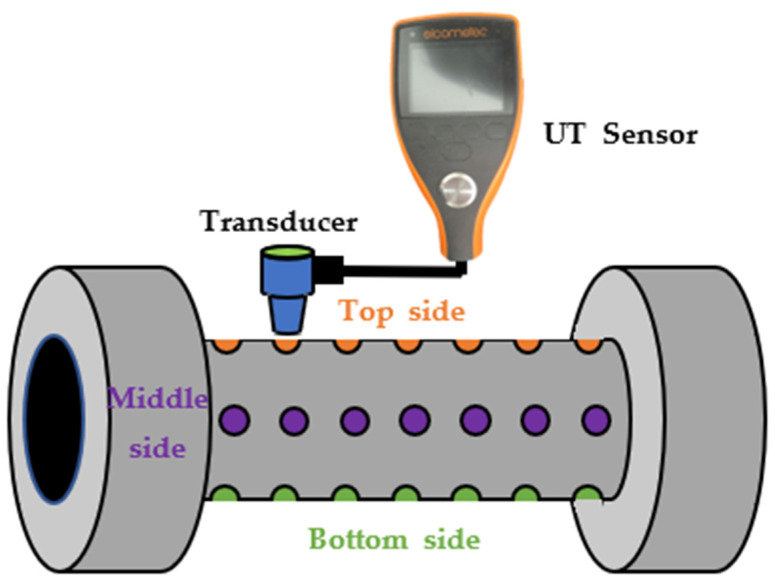
Illustration of remaining thickness measuring points on the test pipe with UT sensor.

A UT sensor (Ultrasonic Thickness Gage, MTG6DL-TXC) was used to measure the remaining thickness of the test pipe made of carbon steel after distinguishing it into top, middle, and bottom sides, as shown in Figure 9. The thickness measurement was repeated for a total of 21 points throughout the experiment. To confirm the accuracy of the measurements obtained from the UT sensor, the average and standard deviation of the measured data were calculated, and the average value of the calculated damaged thickness was compared with the damaged thickness obtained with the ER sensor. After the experiment, the test pipe was disassembled and cut using a fine cutting machine, and the inner surface of the test pipe was observed with a 3D measuring laser confocal microscope (OLS 5000, OLYMPUS, Tokyo, Japan) and a digital camera.

### 3.5. Electrochemical Test

**Figure 10:**
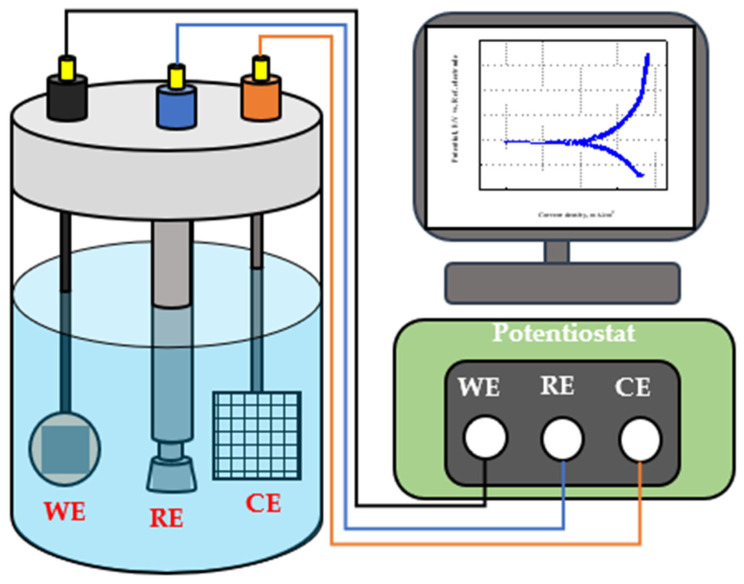
Schematic diagram of three-electrode cell with WE, RE and CE and potentiostat for electrochemical experiments.

An electrochemical experiment was performed to compare the accuracy of commercial corrosion sensors (ER, LPR sensor). A potentiostat (Bio-Logic Science Instrument, FR/VCP) was used for electrochemical experiment, and potentiodynamic polarization and LPR experiments were performed to calculate the *E_corr_*, *i_corr_*, and CR. As shown in Figure 10, the electrochemical cell was composed of a three-electrode cell consisting of a working electrode, a counter electrode (platinum mesh, 2 cm × 2 cm), and a reference electrode (Ag/AgCl electrode saturated by KCl). The working electrode used carbon steel, which is the same material as the test pipe of the test bed. It was mounted with epoxy after cutting to a size of 1 cm × 1 cm. To make the surface of the working electrode as smooth as possible, the surface was polished using 2000 grit SiC paper. The test solution was the same as the fluid used in the test bed, and the test conditions are summarized in Table 2. The potentiodynamic polarization experiments were conducted at a scanning rate of 1.0 mV/s with applied potential from −0.25 to 0.7 V based on the open-circuit potential (OCP) after a rest time of 900 s. The LPR experiment was performed at a scanning rate of 10 mV/min from −30 to 30 mV based on OCP after the rest time of 900 s.

**Figure 11:**
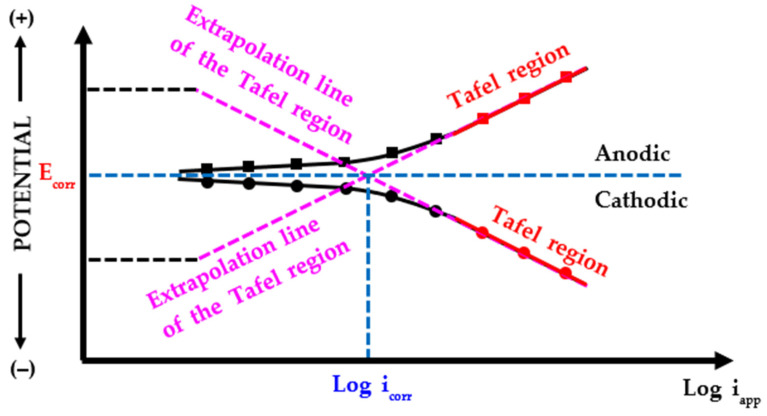
Determination of corrosion potential and current density using the Tafel extrapolation method on the Evans diagram for the oxidation and reduction reactions of metals in corrosive environment.

To calculate the *E_corr_* and *i_corr_* after the potentiodynamic polarization experiment, the Tafel extrapolation method was employed in the range of ±0.25 V based on OCP, as shown in Figure 11. The Tafel extrapolation method measures the *E_corr_* and *i_corr_* using a cathodic or anodic polarization curve based on the mixed potential theory. In the obtained curve, when the current density increases, a region changing to a straight line appears (i.e., a straight region on polarization curve). This is called a Tafel region, and the *E_corr_* and *i_corr_* can be measured by extrapolating the Tafel region and using the intersecting point. The CR can be calculated by applying the measured *i_corr_* to Faraday’s law [40].

After the LPR experiment, the *E_corr_*, *i_corr_*, and *R_p_* were calculated using an analysis tool (EC-Lab software V11.20, Bio-Logic, USA). LPR techniques measure the *E_corr_* and *i_corr_* using electrochemical principles. Compared to the potentiodynamic polarization experiment, LPR can perform measurements within a short time and in the low overpotential region within ±20 V from the *E_corr_*. The relationship between potential and current density is linear in Equation (9).

(9) $$R_{p}=\frac{\Delta E}{\Delta i}=\frac{\beta_{a}\beta_{c}}{2.3\;i_{corr}\left(\beta_{a}+\beta_{c}\right)}$$

Polarization resistance measurement requires the determination of anodic and cathodic Tafel constants, which are normally given in the range of ±20 mV for most metals and alloys. In this study, we determined the Tafel constants within ±20 mV because any error generated from Tafel constants can be neglected when evaluating the relative corrosion rate. To compare the CRs according to each experimental method, the CR was calculated using the *i_corr_* obtained by the LPR method [41].

## 4. Results and Discussion

### 4.1. ER Sensor and LPR Sensor

#### 4.1.1. ER Sensor

##### Effect of NaCl Concentration

**Figure 12:**
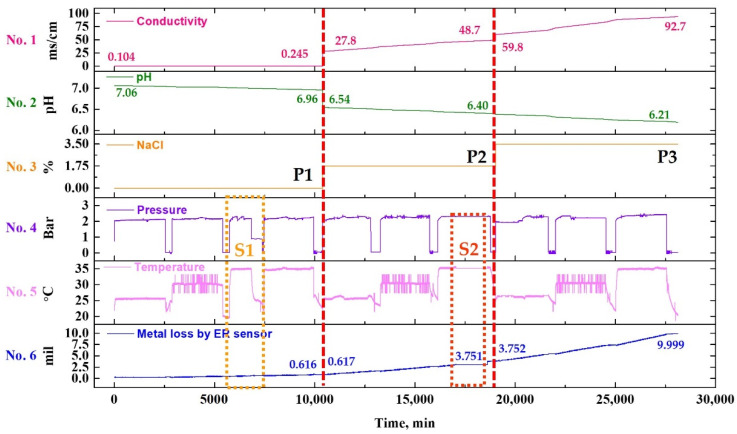
Plot of raw data obtained from different sensors and metal loss from ER sensor.

Figure 12 illustrates a graph showing the data obtained using a conductivity meter, pH meter, pressure sensor, and temperature sensor for comparison with the metal loss value measured by the ER sensor.

In Figure 12, the electrical conductivity and pH data of stacks 1 and 2 are shown by interpolating the data that were measured after collecting the fluid sample at regular intervals during the experiment. Stack 3 is illustrated using the NaCl concentration as the experiment variables, and stacks 4, 5, and 6 are graphs showing the data that were converted to the corresponding value after acquiring the current signal in real-time using the sensor installed in the test bed.

As shown in Figure 12, in the NaCl 0% test condition, both conductivity and metal loss increased with an increase in experiment time and fluid temperature, whereas the pH decreased. This is attributed to the dissolution of metal and particles in the fluid due to damage to the pipe. Generally, the metal ions are desorbed into the solution by an electrochemical reaction (corrosion), such that the conductivity increases and the pH of the solution is lowered by the hydrolysis reaction. This phenomenon was more accelerated when NaCl is added to the fluid. In the case of the second and third programs, 1.75 and 3.5% of NaCl was added, respectively. Consequently, the conductivity, pH, and metal loss graph exhibited rapid fluctuations.

##### First Failure Scenario

**Figure 13:**
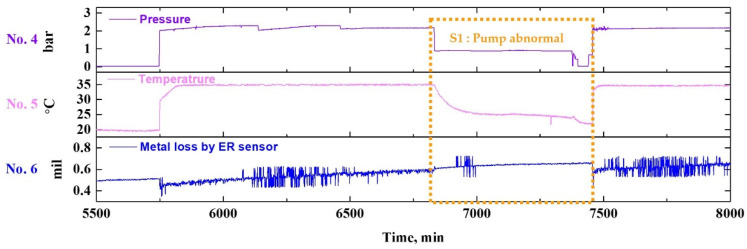
(Extracted from Figure 12) Enlarged view of data showing the sensor responses during the first failure scenario (S1).

Figure 13 shows the results of extracting the section corresponding to the first failure scenario in Figure 11, depicting the state of the ER sensor when the piping system is empty due to failure or ongoing repair. As shown in Figure 13, the ER sensor operates normally, without errors, even if the pipe is empty. In particular, this showed a similar tendency to the state in which the fluid that does not flow was filled. Further, normal operation was achieved even when experiments were performed consecutively without special maintenance. In conclusion, the ER sensor does not affect the operating state of the sensor itself, even if it is not immersed in the fluid.

##### Effect of Fluid Flow

**Figure 14:**
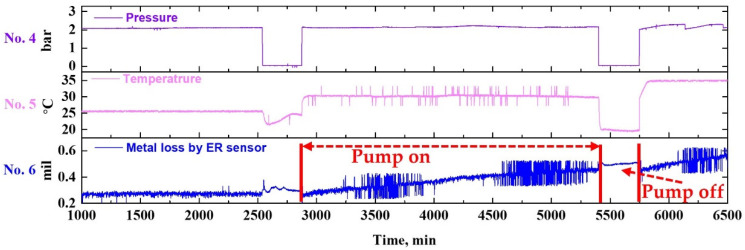
(Extracted from Figure 12) Data showing the effect of the fluid flow on the pipe wall-thinning under the test condition of NaCl 0%.

**Figure 15:**
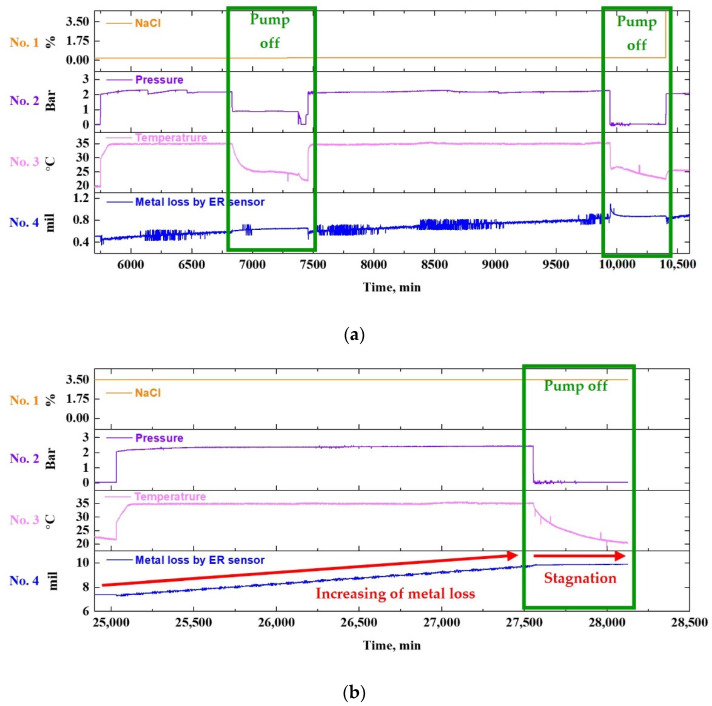
(Extracted from Figure 12) Data showing synergistic effect of NaCl and fluid flow in the presence and absence of NaCl: (**a**) NaCl 0% condition, (**b**) NaCl 3.5% condition.

Figure 14 shows the effect of the fluid flow on the pipe wall-thinning after extracting the range corresponding to the test conditions of NaCl 0%. The metal loss trends were analyzed for the run or stoppage period. When the pump of the piping system was run, the metal loss increased by about 0.213 mil. During the pump stoppage, the metal loss was increased by about 0.018 mil. Using the metal loss values, the CRs can be calculated for pump run and stoppage period, and were 44.594 MPY and 34.698 MPY, respectively. A higher CR was noted for the pump run conditions, which might be attributed to erosion being more dominant than corrosion under the test conditions of NaCl 0%. In addition to this, when NaCl, which accelerates metal corrosion, is present in the fluid, the effect is thought to be more significant [42,43]. In addition, as the thickness of the diffusion layer for dissolved oxygen present in the fluid decreases, the CR of the piping system increases [44,45,46]. To examine the possible synergistic effect of the fluid flow and the presence of NaCl on the metal loss, the following two conditions were compared: NaCl 0% at 35 °C and NaCl 3.5% at 35 °C. The two sets of data are shown in Figure 15.

##### Synergistic Effect of NaCl and Fluid Flow

As shown in Figure 15a, in the case of NaCl 0%, there was little difference in metal loss due to the on/off action of the piping system. This could be attributed to the corrosion reaction being insignificant in the NaCl 0% environment. In contrast, in the case of NaCl 3.5% in Figure 15b, which is a severely corrosive environment, the fluid flow showed an opposite trend in terms of metal loss: a gradual increase when the piping system is in operation, and a rapid decrease when the fluid flow is stopped.

##### Corrosion Rate with Test Parameters

**Figure 16:**
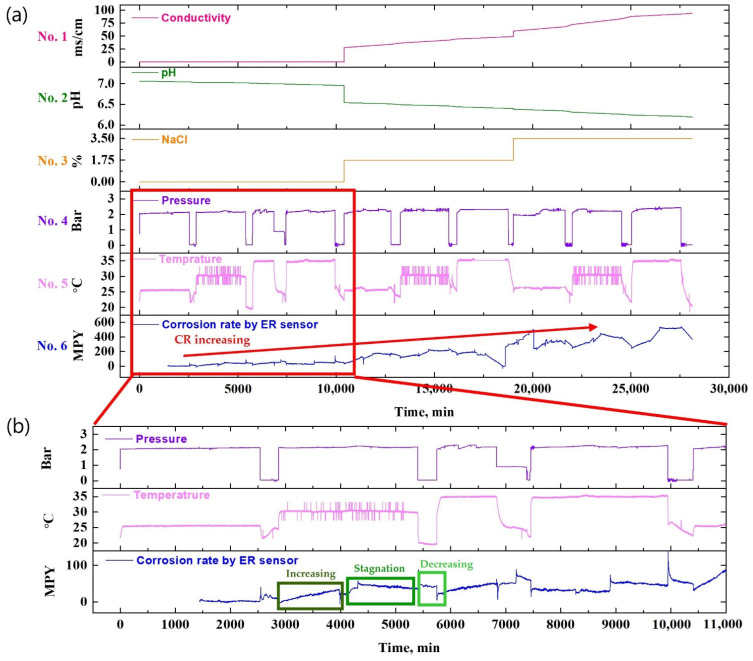
Sensor data including corrosion rate calculated from ER sensor: (**a**) effects of fluid properties on corrosion rate, (**b**) evolution of corrosion rate during the test.

Figure 16a illustrates the comparison of the CR with the test parameters after calculating the former using the metal loss data acquired with the ER sensor. For the data reliability, the CR was calculated for the variance in metal loss corresponding to a period of 1440 min. Overall, as the experiment progressed, the CR increased and decreased depending on whether the pump was operated. The CR appeared to rise with NaCl. Figure 16b details the CR trends in the early stage of the test for further analysis. The analysis results indicate that if the change in the variables is not significant (only the pH and conductivity are changed), the CR increases to a specific value and then stabilizes or stagnates. This exhibits a similar tendency to the corrosion of general metals, which have the stagnant or stabilizing characteristics of the CR, while forming an oxide film on the surface with its initial increase. Vasyliev studied the CRs of metals in the presence of flow rates [47]. The experimental results show that the CR of metals exposed to a corrosive environment tended to decrease over time, and a constant CR is maintained over a certain period. This is in good agreement with the results derived in this study. 

##### Second Failure Scenario

**Figure 17:**
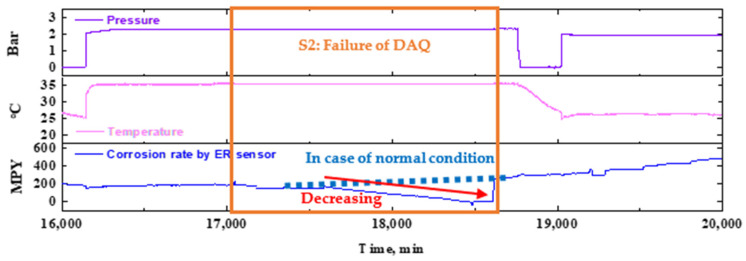
(Extracted from Figure 16) An enlarged view of data showing the sensor responses during the second failure scenario (S2).

Figure 17 illustrates an enlarged graph of the corresponding section, which analyzes the change in the CR during the failure of the DAQ, which represents the second failure scenario. The DAQ stores the last records right before the failure, as if the sensor signals remain active even when communication fails between the DAQ and sensors (Figure 12). There was no change in the metal loss data during the period, so the CR decreased. However, when the communication is restored, past data are ignored, and present data are immediately acquired. Furthermore, if a line is drawn between two labeled points (a) and (b), the missing data can be robustly interpolated (Figure 17). Consequently, the ER sensor is expected to have relatively high reliability for use in piping system monitoring because it recovers the previous state immediately, despite DAQ-related issues.

##### Effect of Fluid Temperature

**Figure 18:**
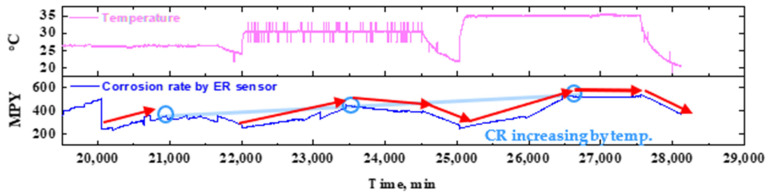
(Extracted from Figure 16) An enlarged view of data showing the variation in corrosion rate with fluid temperature.

Figure 18 shows the graph used to analyze the CR trend as fluid temperature changes. The CR first gradually increased as the experiment was conducted at a constant fluid temperature, and then stabilized over time. This trend (arrow in red) was repeated throughout the test period, indicating that the CR showed a cyclic variation, with an initial increase followed by a subsequent stabilization. The temperature variation led to a change in the corrosion sensor response. As a result, the CR gradually increased with the fluid temperature (line in pale blue). The analysis clearly demonstrates that both the temperature and NaCl concentration of the fluid, as well as the flow rate, have a significant effect on the CR of the piping system.

##### Determination of Corrosion Rate for Each Individual Test Condition

Table 3 shows the wall-thinning and CR corresponding to the test conditions after converting the ER sensor data that were obtained during the experiment into the wall-thinning amount.

The wall-thinning amount and CR determination using the ER sensor indicate that the wall-thinning amount and CR increased with the increase in the temperature and NaCl concentration. In addition to the flow of the fluid, its temperature likewise accelerates metal thinning and the CR using NaCl [35]. In the case of NaCl of 0%, the wall-thinning increased by a factor of 11 times (0.037 mil → 0.424 mil) as the temperature rose. In contrast, in the case of NaCl of 1.75 and 3.5%, the wall-thinning increased by 1.5 (0.816 mil → 1.271 mil) and 1.6 times (1.581 mil → 2.539 mil), respectively. Thus, when NaCl is not present, the influence of temperature on the wall-thinning is relatively significant, but when NaCl is present, the effect of temperature is rapidly reduced. This suggests that temperature is a main factor influencing the wall-thinning when chloride is not present in the fluid. The temperature effect was less pronounced in the chloride environment, since chloride plays a vital role in the corrosion of ferrous metals.

Further, the CR was calculated using the wall-thinning values and time. The obtained CR was less than 100 MPY under the condition of 0% NaCl, while the obtained CR was greater than 150 MPY under NaCl concentrations of 1.75 and 3.5%. It was noticeable that the CR increased by about 5.3 times (42.588 MPY → 223.409 MPY) when NaCl concentration increased from 0% to 1.75%. This trend became saturated even when NaCl concentration doubled from 1.75% to 3.5%, showing that CR increased by 1.87 times (223.409 MPY → 2418.688 MPY). The increase in the CR was greatest at the experiment temperature condition of 25 °C.

##### Comparison of Temperature and Chloride Concentration Effects

**Figure 19:**
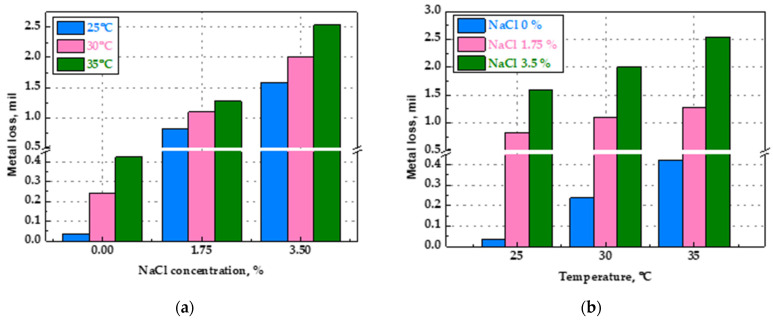
Comparison of metal loss of ER sensor under different NaCl concentrations and fluid temperatures: (**a**) NaCl concentration, (**b**) fluid temperature.

Figure 19 illustrates the graph describing the wall-thinning data according to the NaCl concentrations and the fluid temperatures in Table 3, to analyze their correlation and influence on pipe wall-thinning. Figure 19a shows the increase in metal loss with the fluid temperature variables in each NaCl condition, whereas Figure 19b shows the rise in metal loss with NaCl concentrations under the fluid temperature condition. Under an increased NaCl concentration, the changes in metal loss were much greater than those induced by increased fluid temperature. This indicates that the NaCl concentration is a more determinant factor for metal loss compared with fluid temperature.

#### 4.1.2. LPR Sensor

**Figure 20:**
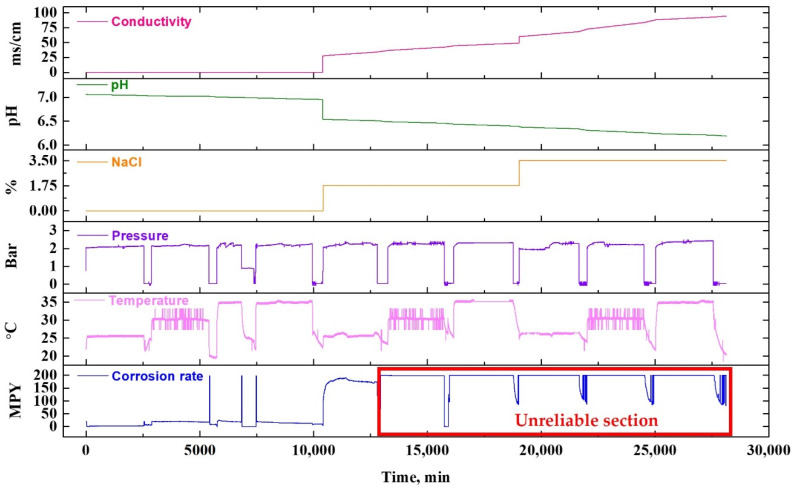
Plot of raw data obtained from different sensors and the corrosion rate calculated from LPR sensor.

Figure 20 compares the data obtained through each type of sensor with the LPR sensor data. The current signal was obtained through the LPR sensor, which results from the calculation of the CR.

As shown in Figure 20, under the NaCl 0% test condition, when the fluid temperature is 25 °C, 4 MPY is obtained, and as the fluid temperature increases, the CR is measured to be about 20 MPY. In contrast, in the case of a solution containing 1.75% NaCl, about 190 MPY was measured at 25 °C, and 200 MPY was measured at 30 °C or higher. In the case of the LPR sensor used in this study, the measurement range is 0–200 MPY. Therefore, CRs measured above 200 MPY are unreliable, including the measured NaCl 1.75% and fluid temperature of 30 °C. As described above, the LPR sensor is convenient, as the signal output during measurement indicates the CR. Nevertheless, it has limitations, as it cannot be used in a severe corrosion environment due to its fixed measurement range.

#### 4.1.3. Comparison of Corrosion Rate by the Corrosion Sensor and the Electrochemical Experiment (Lab Scale)

**Figure 21:**
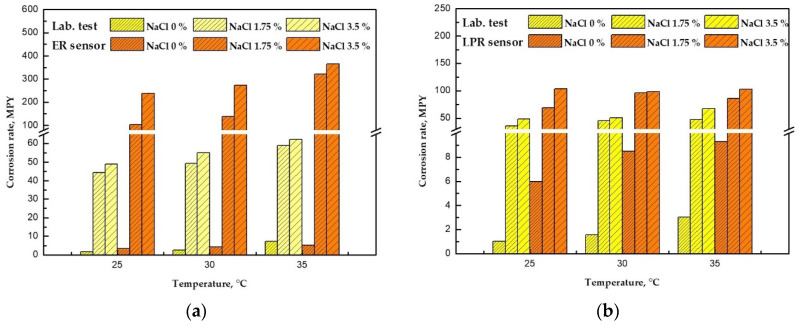
Comparison of corrosion rates obtained different methods and experimental conditions: (**a**) ER sensor versus potentiodynamic polarization experiment (lab. test) and (**b**) LPR sensor versus linear polarization resistance (lab. Test).

Figure 21 compares the CRs obtained using ER and LPR sensors, and the CR calculated by electrochemical experiment. The lab-scale electrochemical experiments, including potentiodynamic polarization and linear polarization, were conducted under the same test conditions as the corrosion sensors. The results of the experiment are described in Figures S1–S5 in the Supplementary Materials.

Considering the similarity of the experimental methods, the ER sensor and the potentiodynamic polarization experiment were compared, whereas the LPR sensor was compared with the linear polarization resistance experiment. Furthermore, the CRs measured during the stoppage of the piping system were compared. As shown in the graph, in the absence of chloride, the CR measured using the ER and LPR sensors was similar to the CR measured using the electrochemical experiment. However, in the presence of chloride, the ER and LPR sensors showed a significantly higher CRs. Thus, the CR measured using a commercial corrosion sensor and the CR calculated using the lab scale may differ depending on the various experimental parameters. Therefore, corrosion damage can be further accelerated using various types of corrosion and environmental variables, such as galvanic corrosion and stress corrosion cracking. Fluid pressure, which may occur in the operating environment of the actual piping system, may also cause corrosion fatigue. Therefore, UT thickness measurements were used to find a method that better matches the CR of the real piping system.

### 4.2. UT Measurements

#### 4.2.1. Result of UT Thickness Measurements of Test Pipe

**Figure 22:**
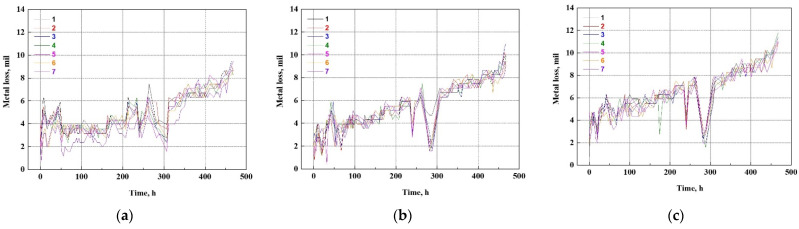
Variations in metal loss with time converted from the remaining thickness of test pipe measured by UT sensor for three different regions of the test pipe: (**a**) top side, (**b**) middle side and (**c**) bottom side.

The remaining thickness of the test pipe was determined using UT thickness measurements to compare the accuracy of the corrosion damage and rate obtained from the corrosion sensor and at the lab scale. The measured values were converted to a metal loss compared with the damaged thickness caused by the ER sensor. The damaged thickness of the test pipe increased with the test time at all measurement points (Figure 22). Particularly, the increased damaged thickness was shown in the order of bottom > middle > top side. As stated in the introduction, the measurement of the remaining thickness of the test pipe does not always indicate a constant trend, depending on the operator’s skill level. Nevertheless, the final measured value showed a similar result to the thickness reduction measured using the ER sensor.

#### 4.2.2. Internal Surface Analysis of Test Pipe after the Corrosion Experiment

To analyze the degree and tendency of damage according to the location of the test pipe, the test pipe was cut after the test, and the surface was examined with a 3D microscope. A summary of the analysis using a 3D microscope is provided in Figure S7.

**Figure 23:**
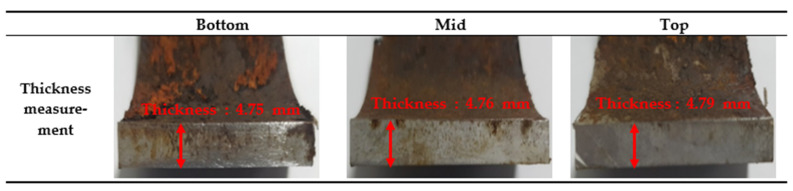
Actual remaining thickness measurements for three different test pipe positions after experiment.

Figure 23 shows the results of the remaining thickness, measured after cutting the test pipe. For accurate thickness measurements, the oxides formed on the surface were removed using a physical method. Carbon steel generally forms corrosion oxides or hydrates on the surface through electrochemical reactions with various ions, such as oxygen and chloride. The corrosion oxides formed on the surface exhibit passivation characteristics and inhibit the corrosion of metals from solutions. These oxides are known as oxide or passivation films. However, corrosion is not protected, as the oxide film is not densely formed in environments where chloride is present. As a result of 3D microscopic analysis in Figure S7, corrosion oxides or hydrates were observed at a relatively high density at the bottom side of the test pipe, and the surface roughness value was similarly large.

In contrast, the lowest amount of corrosion oxide or hydrate was observed on the top side, and the surface roughness value was the smallest. Thus, the tendency of corrosion oxide and hydrate formation is different depending on the point at which the pipe makes contact with the fluid. The density of the corrosion product is considered to be high, as corrosion oxides and hydrates are formed in the upper direction due to the positional characteristics of the bottom side, and the desorption phenomenon is relatively small. However, the density of the top side is considered to be relatively low as corrosion oxides and hydrates grow in the downward direction, and desorption of the product occurs more actively. When measuring the remaining thickness of the test pipe, the remaining thickness of the bottom side was measured to be the smallest, and the top side was estimated to be the largest.

#### 4.2.3. Statistical Analysis; Average, Standard Deviation, and Polynomial Regression

**Figure 24:**
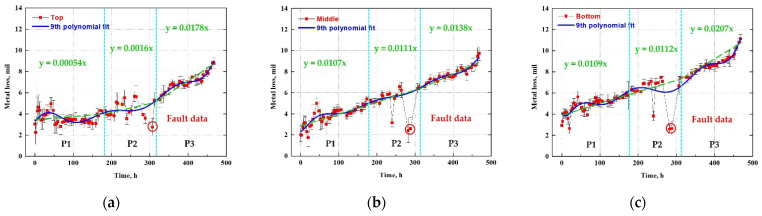
Polynomial regression analysis of metal loss data obtained from UT sensor after calculating average and standard deviation: (**a**) top side, (**b**) middle side and (**c**) bottom side.

Figure 24a–c plot the average and standard deviation of the data to reveal the deviations and trends in the UT measurement data at different measurement positions. The solid line in a green color is the ninth-order polynomial fitting using a commercial graphing software (Origin Pro 9), and the dotted line in purple color indicates the linear fitting of the damaged thickness corresponding to the range of chloride concentration variables. The curve fitting with a ninth-order polynomial revealed that the final damaged thickness had a similar tendency to the corrosion sensor as the experiment time, and chloride concentration increased. In particular, as a result of the linear fitting for each chloride concentration section, the slope of the damaged thickness graph was found to increase with the chloride concentration.

#### 4.2.4. Linear Regression Analysis and Adjusted Coefficient of Determination

**Figure 25:**
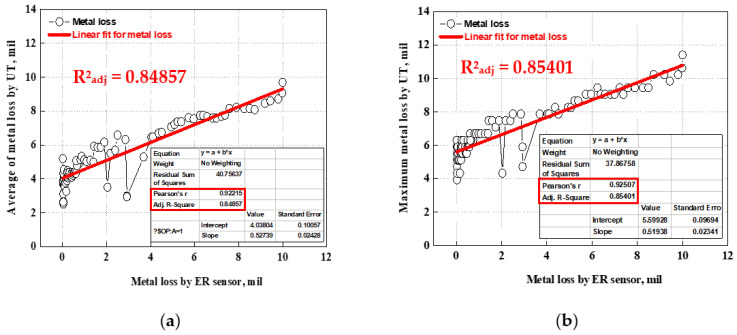
Linear regression analysis and adjusted coefficient determination for metal loss by ER and UT sensor: (**a**) metal loss versus average metal loss by UT sensor and (**b**) metal loss versus maximum metal loss by UT sensor.

As discussed, a measurement error is inevitably produced when measuring the pipe thickness using the UT sensor. In this study, however, thickness measurements by UT sensors indicated a tendency to follow the wall-thinning trend, and the terminal metal loss corresponded to the actual pipe thickness in Figure 23. Based on these results, a regression analysis was performed between the damage thickness measured by the ER sensor and UT measurement data, as shown in Figure 25. To pre-process the UT measurement data, the data were calculated as the average and maximum damage values of the data measured at all measurement points on the upper, middle, and bottom sides at the same time, and are described in Figure 25a,b.

This analysis shows that the R^2^_adj_ value (adjusted coefficient of determination) was about 0.848 and 0.854, respectively, which can be interpreted as a similar trend existing between the ER sensor and UT measurement data. Consequently, it is possible to apply a commercial ER sensor to evaluate the remaining thickness of the piping system, and this is considered to have high accuracy when calculating the CR.

### 4.3. Correlation Coefficient Analysis of Metal Loss and CR Considering Various Factors

Correlation analysis is used to determine the correlation or intensity of correlation between two continuous variables [48]. The intensity of correlation can be quantified by calculating a correlation coefficient. Therefore, the correlation between two variables can be determined using the calculated correlation coefficient, and the correlation intensity can be evaluated to rank correlations for various variables [49]. The Pearson correlation coefficient, Spearman correlation coefficient, and Kendall’s tau correlation coefficient are typically used as ways to calculate the correlation coefficient [50]. 

**Figure 26:**
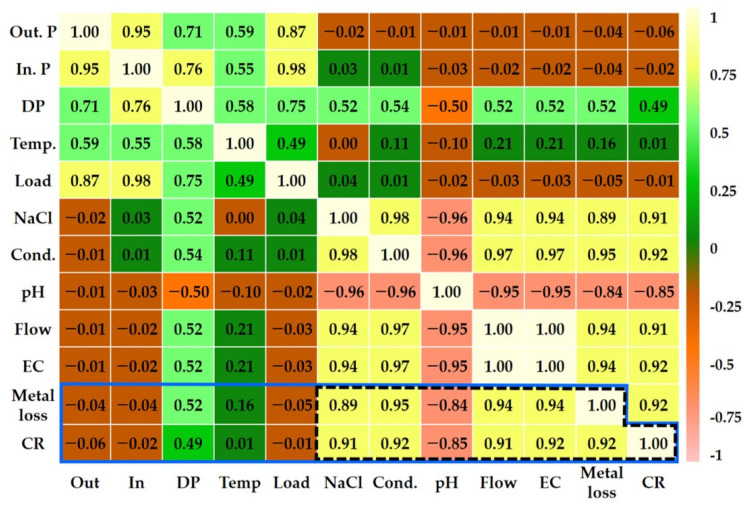
Pearson correlation coefficient map for correlation analysis between metal loss (corrosion rate) and various fluid parameters. The Pearson correlation coefficient indicating strong correlation (≥±0.8) is marked with a black dashed line.

In this study, the Pearson correlation coefficient was employed to identify the variables that correlate with the features of metal loss and CR. The calculated results are shown in Figure 26. Two datasets were integrated using time as a pre-processing process so that the obtained pump pressure and pump motor load data were among the experimental variables used in this study for Pearson correlation coefficient analysis. Moreover, using the calculated flow and quantity of electric charge, the correlation coefficient was calculated and the correlation was analyzed. The lighter color indicates a significant correlation coefficient, and the darker color indicates a small correlation coefficient. Generally, a correlation coefficient value greater than ±0.8 indicates a strong correlation.

As shown in the pink area of Figure 26, NaCl, conductivity (cond.), pH, flow, and quantity of electric charge (EC) were found to have a strong correlation with metal loss and CR. In particular, as the pH decreased, the metal loss and CR showed a negative correlation coefficient, and the remaining variables had a positive correlation coefficient.

## 5. Discussion

### 5.1. Various Factors Affecting Corrosion and Wall-Thinning of Piping System

One of the primary concerns for metallic pipelines involve internal damage from corrosion, leading to wall-thinning. There are several different environmental factors affecting corrosion, including temperature, conductivity, pH, flow rate and fluid pressure. In this study, NaCl concentrations, fluid flow and temperature were considered to be the most significant factors regarding the corrosion of the ER sensor, which is the identical material used in the piping system.

As shown in Figure 12, metal loss in the ER sensor increases with NaCl concentrations. NaCl is a major factor accelerating the electrochemical corrosion reaction of metals. In particular, it accelerates corrosion damage by self-propagating and autocatalytic mechanisms, as the hydration reaction of chloride ions lowers the pH. Therefore, when NaCl is added, the metal loss of the ER sensor undergoes a rapid increase.

The fluid flow is another important factor affecting the internal damage to a pipe. In general, the fluid flow accelerates wall-thinning by affecting pipe erosion and corrosion reactions. First, pipe erosion refers to a phenomenon that causes the continuous wall-thinning of metal pipes due to mechanical and physical interactions between the fluid flow and the metal pipe surface [51]. Furthermore, the mechanism of erosion that can occur in the piping system varies according to the pressure changes and erosion behavior of the fluid, such as cavitation erosion, flashing erosion, liquid impingement erosion, and solid particle erosion [52]. Second, the corrosion reaction accelerates the wall-thinning of the piping system, as the electrochemical reaction rate changes according to the fluid flow. This phenomenon is known as flow-accelerated corrosion (FAC) and has been studied by numerous researchers. The FAC mechanism is as follows [53]. Generally, the thickness of the mass transfer boundary layer is reduced when there is fluid flow. This facilitates the diffusion of Fe^2+^ ions and generation of corrosion products due to the corrosion reaction of the metal, leading to an increase in the CR of the metal [54,55]. In the particular case of carbon steel, which cannot form a relatively strong and dense passivation film, the CR significantly increases as the fluid flow velocity increases [56]. Thus, when the flow velocity of the piping system is fast, the corrosion reaction is activated according to the decrease in the thickness of the mass transfer boundary layer between the inner surface of the piping and the solution.

The potential synergistic effects on CR between NaCl concentration and fluid flow were studied. The CR appears differently as the electrochemical reaction rate changes depending on the flow velocity of the piping system, and the flow velocity can be considered to affect the CR of the piping system. In addition, physical damage, such as erosion, may occur if flow velocity is present. However, according to the experimental results in this study, in the absence of NaCl, the damage caused by the flow velocity was insignificant. Combining these results, the increase in the CR has a more significant effect on the damage of the pipe than the effect of physical erosion in the flow velocity conducted in this study. Nevertheless, the damage caused by physical erosion cannot be ignored at higher flow velocities. In particular, when two factors work together rather than each independently affecting the pipe, the wall-thinning of the piping system can be accelerated by exerting a synergistic effect [57]. Therefore, the flow velocity is an essential factor to be considered when evaluating the remaining useful life of the piping system [58,59].

Aside from the NaCl and fluid flow, the temperature also increases the CR measured by the ER sensor (Figure 18). This is because, when the temperature increases, the CR rises by enhancing the activity of the various ions participating in the electrochemical reaction and changing the free energy that determines the degree of reaction between metal and fluid [60]. When the fluid is at room temperature, the effect on metal loss according to the increase in temperature is insignificant, as, in the relatively low-temperature environment, erosion is more dominant than corrosion [61,62]. In the case of the flow velocity applied in this study, the damage due to erosion was minimal, the temperature of the controlled variable was low, and the change rate was not significant, so that the effect of the temperature was considered negligible. In contrast, because NaCl is a major factor accelerating metal corrosion, even a small amount can have a relatively significant effect. From Figure 14 and Figure 19, it can be seen that NaCl, start/stop, and fluid temperature, which are the test variables that were selected in this study, correlate with each other and affect the pipe wall-thinning. However, as the effects of start/stop and fluid temperature on pipe wall-thinning differ depending on the presence or absence of NaCl, that NaCl was evaluated to have the most significant influence.

### 5.2. Reliability of ER Sensor

The reliability of the ER sensor was evaluated by comparing the results obtained from the cross-sectional observation and thickness measurements that were carried out mechanically (ruler) and ultrasonically for the damaged pipe. The cross-sectional observation demonstrated the different amounts of damage produced in the different positions and orientations of the pipe.

The results of the cross-sectional analysis indicated that the bottom side experiences the most damage due to corrosion and erosion. According the results of 3D analysis and thickness measurements, the electrochemical reaction occurs most actively on the bottom side compared to the top, resulting in high-density oxides and hydrates and greater corrosion damage. Corrosion oxides and hydrates accumulate due to the positional characteristics of the bottom side, and the influence of gravity further accelerates erosion and corrosion by fluid.

The remaining thickness measurement showed that the top side had the largest remaining thickness, while the bottom side had the smallest remaining thickness. These results nearly correspond to the UT sensor measurement result shown in Figure 22. Furthermore, the average value of the damaged thickness for the three positions showed similar results to the damaged sensing element thicknesses of the ER and LPR sensors in Figure S6.

The UT measurement method is generally considered to be suitable for measuring the damaged thickness of the piping system. However, as in the ninth-order polynomial fitting, the trends in the measured data is inconsistent, with numerous large deviations. This is because pipe thickness measurements made with a UT sensor may generate different measurement accuracies depending on the surroundings, fluid flow, and operator measurement skill, as stated in the introduction. Given the disadvantages of the UT sensor, the ER sensor should be a more reasonable choice than the conventional UT sensor when determining the metal loss and CR of pipes.

### 5.3. Correlation Coefficient Analysis

The correlation coefficient analysis was carried out between the operating parmeters and ER sensor, and a revealed strong relationship was revealed between the ER sensor and fluid parameters such as NaCl concentration, conductivity, pH, flow and quantity of electric chage. This was consistent with the results that were derived by analyzing the factors affecting the corrosion damage of the piping system according the electrochemical reaction obtained by measuring the metal loss and CR using the ER sensor in Section 4.1. The conductivity and pH are factors affected by NaCl. The pH showed a relatively low correlation coefficient, which can be attributed to the relatively small range of changes due to the characteristics of the pH data. This showed similar characteristics in terms of the variable of the fluid temperature. In general, the temperature is considered to be a major factor influencing the corrosion of metals and aqueous solutions [35,63]. However, because the fluid temperature variable used in this study has a relatively small range, it was not considered to significantly affect the corrosion damage. In contrast, the NaCl concentration and flow showed a relatively large correlation, as they had a variable range that could sufficiently affect the metal corrosion.

## 6. Conclusions

Internal pipe wall-thinning and the CR were measured in various pipe usage environments using a custom-built test bed equipped with various sensors. The characteristics of the CR change were analyzed according to the presence or absence of NaCl concentrations, temperature, and flow velocity, and the results were compared with those of the lab scale electrochemical setup.

The damaged thickness and CR measurements obtained using a commercial ER sensor indicate that the CR increases with NaCl concentrations, fluid temperature, and flow velocity. Notably, the ER sensor’s wall thickness determination strongly correlates with the UT sensor’s measurements. A direct measurement of the remaining thickness of the pipe after the test confirmed that the thickness of the real pipe corresponds to the measurements of both ER and UT sensors. Contrary to expectations, the LPR sensor did not yield the expected CR in a corrosive environment containing NaCl. The LPR sensor reading reached its maximum value under a NaCl concentration of 1.75% at temperatures above 30 °C, indicating that the accuracy of CR may have been compromised. This is attributed to the following measuring methods of the sensor: the ER sensor is based on the physical principle of measuring changes in the electrical resistance due to metal loss, whereas the LPR sensor is based on the electrochemical principles in which the polarization resistance is obtained.

The CR sensing accuracy was evaluated by comparing the CR response from the corrosion sensors and the reference CR obtained from a lab-scale electrochemical experiment. As expected, the results of the electrochemical evaluation showed a remarkably low CR compared to that of the ER sensor. The reason for this is clear: there was little flow velocity in the lab scale experiment, which would not lead to a change in the mass transfer boundary layer between the working electrode and the aqueous solution; thus, the corrosion reaction was suppressed. However, both the ER sensor and electrochemical evaluation demonstrated a similar trend to NaCl concentration and fluid temperature.

The inner surface of the pipe was optically observed after the test to confirm the accuracy of the metal loss in the ER sensor. The remaining pipe thickness was also measured at regular intervals using a UT sensor, and a regression analysis was performed using the metal loss values from the ER and UT sensors. The analysis of thickness variations revealed that the metal loss measured by the ER sensor is in good agreement with both the wall-thinning value of the actual pipe and the value measured by the UT sensor. In the regression analysis, a strong correlation was found between the metal loss values from the ER sensor and those from UT sensors. Consequently, the commercial ER sensor was found to have high reliability to monitor the metal loss and evaluate the remaining pipe thickness.

To analyze the correlation of factors affecting the wall-thinning of the pipe, the correlation coefficient was calculated using the operating parameters and the sensor response from the ER sensor. The NaCl amount and flow showed a high correlation coefficient of 0.9 or more. However, the temperature had a lower correlation coefficient than other variables, possibility due to the limited range of the test.

The results of this investigation demonstrated that the ER sensor has acceptable reliability for monitoring the wall thickness of industrial pipelines. However, the sensing element of 10 mil thickness only had a short lifetime (approximately 2 weeks), although the test was conducted under highly accelerated and harsh conditions for the sensor. Thus, the thickness of the sensing element should be considered under harsh operating conditions, particularly in chloride-containing environments. In addition, there are several other aspects of implementation, such as material, wall thickness, and the coating of the target pipe.

To date, the ER sensor has been used successfully in corrosion monitoring, but few studies have reported any relationship between operating parameters and pipe wall-thickness. The results of this work not only provide useful information for the determination of CR using an ER sensor under various operating parameters in the piping system, in addition to new insights regarding the use of ER sensor as a pipe wall-thickness measuring method, which provides an alternative to the conventional UT sensor.

Further studies are required to follow-up our pilot research: A pipe lifetime prediction algorithm should be developed using a single metallic sensing element to extend its application to the integrity monitoring of various metallic pipes using machine learning. A machine learning model can be trained with the labeled dataset to establish the pipe’s lifetime prediction model. The training data consist of the pipeline operating data as input and the corrosion sensor response (i.e., metal loss or CR) as target output. If the model is trained, it could predict the remaining thickness of the pipeline, which is the output of the model. To explore the feasibility of a long-term of use of the sensor, more resistant metals and alloys, such as stainless steel, can be used as the sensing element. Data from different sensing materials can be correlated, based on the corresponding experimental data. Future work may benefit from the present findings and data.

As presented in this study, the ER sensor is considered to be the most reasonable and suitable sensor for monitoring the wall-thinning of pipes for industrial applications, and can provide an alternative to UT sensor to obtain the pipe’s wall thickness. It should be noted that the implementation of an ER sensor requires a correct understanding of the electrochemical reaction mechanisms that occur under various operating conditions.

## Figures and Tables

**Table 1:** Type of sensors and their properties.

Name of Sensor	Measuring Range	Output Signal
Pressure sensor for strainer inlet	0–100 kPa	4–20 mA
Pressure sensor for strainer outlet	0–100 kPa	
Pressure sensor for pump outlet	0–1 MPa	
Differential pressure sensor for strainer	0–100 kPa	
Temperature sensor for fluid	−50–250 °C	
Current transmitter for pump	0–5 A	
LPR sensor	0–200 MPY	
ER sensor	0–10 mils	

**Table 2:** Experimental conditions for the electrochemical tests to compare results of corrosion rate with the commercial corrosion sensors.

No.	1	2	3	4	5	6	7	8	9
NaCl (%)	0			1.75			3.5		
Temp. (°C)	25	30	35	25	30	35	25	30	35

**Table 3:** Results of calculation of metal loss and corrosion rate with ER sensor with different experimental conditions.

Time (min)	0–10,407			10,408–19,023			19,024–28,127		
NaCl (%)	0			1.75			3.5		
Temp. (°C)	25	30	35	25	30	35	25	30	35
Wall thinning (mil)	0.037	0.239	0.424	0.816	1.092	1.271	1.581	2.001	2.539
Sum of Wall thinning (mil)	0.7			3.179			6.121		
MPY	7.653	49.691	62.424	179.301	232.277	255.366	314.644	419.014	526.845
Average of MPY	42.588			223.409			418.688		

## Data Availability

The data are not publicly available due to the project requirements.

## Supplementary Materials

The following supporting information can be downloaded at: https://www.mdpi.com/article/10.3390/s22197562/s1, References [64,65,66] are cited in the supplementary materials. Figure S1: Potentiodynamic polarization curves of the carbon steel under different fluid temperatures and NaCl concentrations: (a) NaCl 0%, (b) NaCl 1.75% and (c) NaCl 3.5%.; Figure S2: Comparison of electrochemical corrosion parameters of the carbon steel with different fluid temperatures and NaCl concentrations: (a) corrosion potential and (b) corrosion current density.; Figure S3: Linear polarization resistance curves of the carbon steel with different fluid temperatures and NaCl concentrations: (a) NaCl 0%, (b) NaCl 1.75% and (c) NaCl 3.5%.; Figure S4: Comparison of electrochemical corrosion parameters of the carbon steel obtained from the linear polarization resistance with different fluid temperatures and NaCl concentrations: (a) corrosion potential, (b) corrosion current density and (c) polarization resistance.; Figure S5: Comparison of corrosion rates obtained from different electrochemical evaluation methods under different fluid temperatures and NaCl concentrations: (a) potentiodynamic polarization and (b) linear polarization resistance.; Figure S6: Photographs of (a) sensing element of ER sensor and (b) sensing element of LPR sensor after the test.; Figure S7: Surface analysis of the inner side of the test pipe using 3D laser confocal microscope.; Table S1: Results of calculation of corrosion rate after potentiodynamic polarization and linear polarization resistance experiment.
