# Supplementary material for: Evaluation of Commercial Corrosion Sensors for Real-Time Monitoring of Pipe Wall Thickness under Various Operational Conditions
Source: Sensors (Basel). 2022 Oct 6;22(19):7562. doi: 10.3390/s22197562 (PMC9571097; doi:10.3390/s22197562)
Supplement: Supplementary file 1 [file sensors-22-07562-s001.zip › sensors-1927141-supplementary.pdf]

Supplementary materials

Potentiodynamic polarization test

Figure S1 shows the results after performing a potentiodynamic polarization experiment under the same conditions for the corrosion sensors. As the NaCl concentration and temperature increased, the current density increased at all potentials of the potentiodynamic polarization curve. In the composition of the carbon steel used in the test, iron (Fe) occupies the largest proportion. Iron exhibits the characteristics of forming an oxide film (passive film) that protects the base metal by creating oxides Fe_2O_3 and Fe_2O_4 by oxidation reactions in the solution saturated with oxygen [35]. However, if chloride is present in the solution or the temperature is increased, the oxide film formed is not strong and thus is easily destroyed, further accelerating the corrosion damage to the base material [64,65].

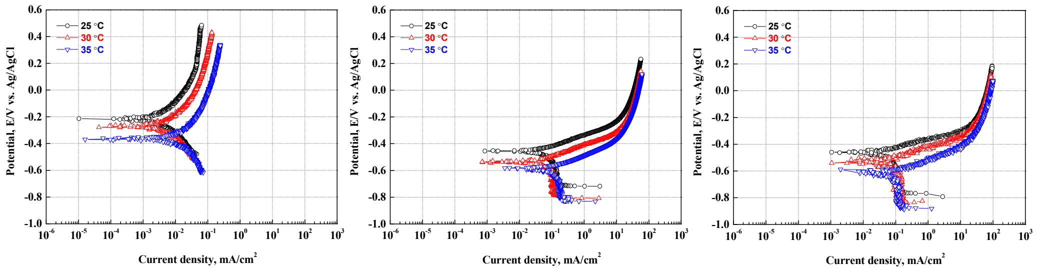

(a) (b) (c)

Figure S1. Potentiodynamic polarization curves of the carbon steel under different fluid temperatures and NaCl concentrations: (a) NaCl 0%, (b) NaCl 1.75% and (c) NaCl 3.5%.

In Figure S2, the Tafel extrapolation method yields the E_{corr} and i_{corr}. The E_{corr} exhibited active against the reference electrode under all test conditions. Further, as the chloride concentration and temperature increased, the E_{corr} decreased, and the i_{corr} increased. In particular, the E_{corr} and i_{corr} changed significantly depending on the presence or absence of NaCl. Similar to the experimental results using the corrosion sensor in the test bed, the NaCl concentration was found to have a more significant effect on the CR of the metal pipe than the increase in temperature.

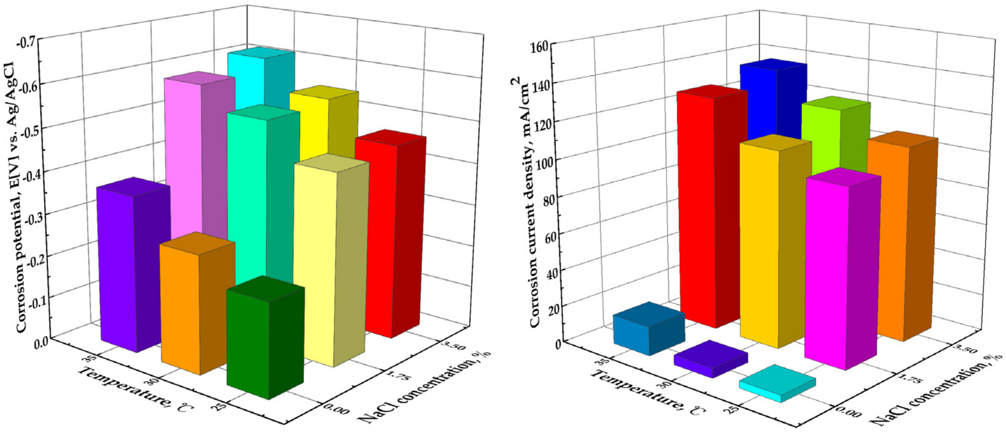

(a) (b)

Figure S2. Comparison of electrochemical corrosion parameters of the carbon steel with different fluid temperatures and NaCl concentrations: (a) Corrosion potential and (b) Corrosion current density.

Linear polarization resistance test

Figure S3 shows the results of the LPR experiment under the same test conditions as those of the potentiodynamic polarization. In order to compare the results with different conditions, linear fitting was performed for each potential-current density curves. The linear fits included the over-voltages ranging from – 20 mV to 20 mV (vs. E_{corr}), which is regarded as a typical range of voltages showing general linearity during linear polarization resistance test.

Similar to the results of the potentiodynamic polarization experiment, the slope of the curve decreased as the chloride concentration and temperature increased. This means that as the chloride concentration and temperature increase, the ratio of the current density to the potential rises.

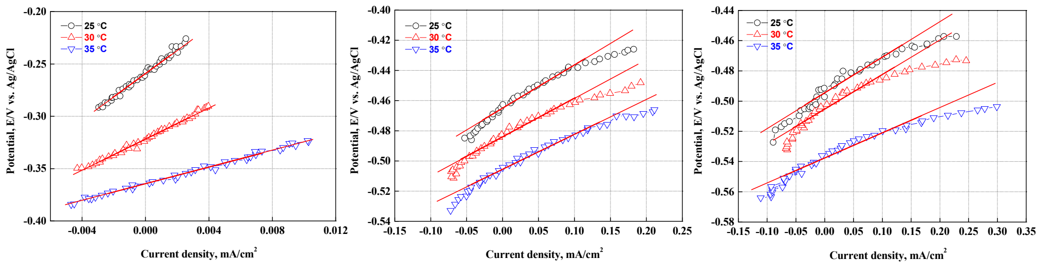

(a) (b) (c)

Figure S3. Linear polarization resistance curves of the carbon steel with different fluid temperatures and NaCl concentrations: (a) NaCl 0%, (b) NaCl 1.75% and (c) NaCl 3.5%.

The E_{corr}, i_{corr}, and R_p were calculated using an analysis tool in Bio-logic's electrochemical analysis software for comparison with the results of the potentiodynamic polarization experiment. The results are shown in Figure S4. The E_{corr} and i_{corr} were similar to the results of the potentiodynamic polarization experiment. In the case of R_p, as opposed to i_{corr}, the E_{corr} decreased with increasing chloride concentration and temperature. The decreased R_p indicates that the interfacial resistance between the working electrode (carbon steel) and the test solution decreased, resulting in an active electrochemical reaction and accelerated corrosion damage.

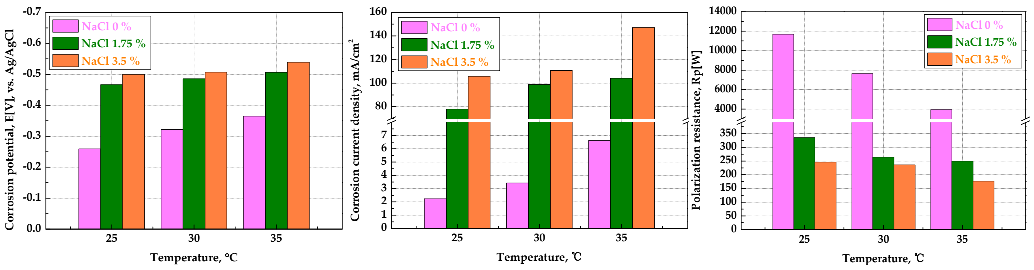

(a) (b) (c)

Figure S4. Comparison of electrochemical corrosion parameters of the carbon steel obtained from the linear polarization resistance with different fluid temperatures and NaCl concentrations: (a) Corrosion potential, (b) Corrosion current density and (c) Polarization resistance.

Comparison of corrosion rate calculated after the electrochemical experiment

Figure S5 and Table S1 present the results of calculating the CR using the icorr calculated after the potentiodynamic polarization experiment and the LPR experiment based on ASTM-G102. The formula for the CR is as follows [37].

$$CR = K_1 \frac{i_{corr}}{\rho} EW$$

$K_1 = 3.27 \times 10^{-3}$, mm g/μA cm yr

i_{corr} = Corrosion current density

ρ = Density in g/cm^3

EW = Equivalent weight

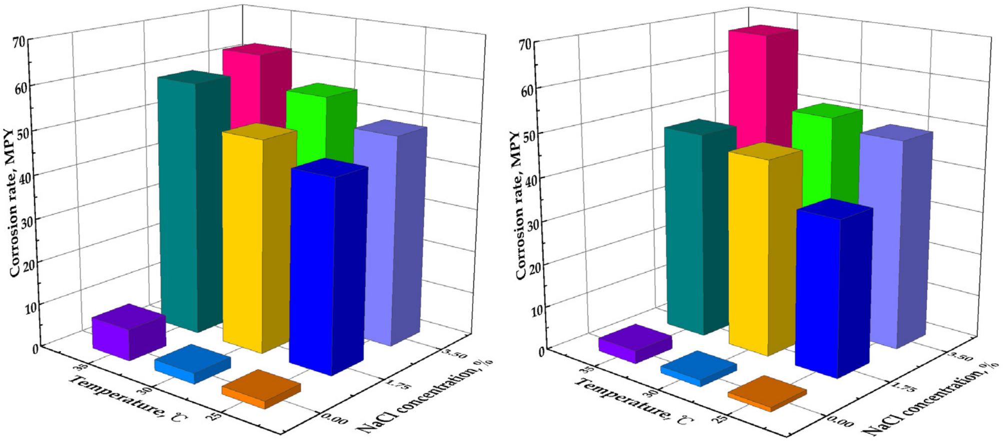

(a) (b)

Figure S5. Comparison of corrosion rates obtained from different electrochemical evaluation methods under different fluid temperatures and NaCl concentrations: (a) Potentiodynamic polarization and (b) Linear polarization resistance.

Table S1. Results of calculation of corrosion rate after potentiodynamic polarization and linear polarization resistance experiment.

Test	CR by Potentiodynamic polarization			CR by Linear Polarization Resistance		
NaCl / Temp.	0 %	1.75 %	3.5 %	0 %	1.75 %	3.5 %
25 ℃	1.7	44.2	48.9	1.0	35.7	48.4
30 ℃	2.4	49.1	54.9	1.5	45.2	50.7
35 ℃	7.3	58.7	62.2	3.0	47.7	67.3

The two graphs showed a similar trend: the CR increased with the chloride concentration and temperature. In particular, the CR changes rapidly depending on the presence or absence of chloride.

Observation of ER and LPR sensor after corrosion experiment

Figure S6 compares damaged sensing element of ER (a) and LPR (b) sensors after experiments with new ones. After the experiments, an electrochemical reaction of both sensors formed a red oxide. In particular, the ER sensor showed that the sensing element with a thickness of 10 mils (254 μm) was completely destroyed by the corrosion reaction. Further, the thickness of the LPR sensor probe was reduced due to the corrosion reaction. The oxide formed on the probe surface was removed for a more accurate remaining thickness measurement, according to the protocol ASTM G1-03 (Standard Practice for Preparing, Cleaning, and Evaluating Corrosion Test Specimens), which removed the oxide by immersing the sensing element of LPR sensor into the prepared removal solution [66]. The measured remaining thicknesses of the sensing element of LPR sensor after removing the oxide were 4.34 mm and 4.27 mm, respectively. Compared to the new standard thickness of 4.75 mm, the thicknesses were reduced by 0.41 and 0.48 mm, respectively. In the case of measuring the remaining thickness of the sensing element of LPR sensor, as both sides were damaged at the same time, half of the thinned thickness can be understood as the actual thickness reduction. Consequently, when compared with the ER sensor, the sensing element of the LPR sensor, which was tested at the same time, showed a value that was almost the same as the damaged thickness.

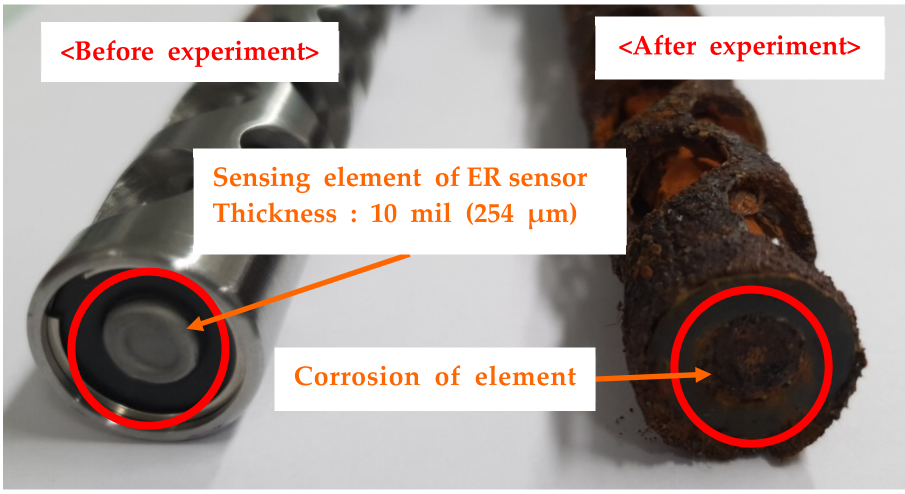

(a)

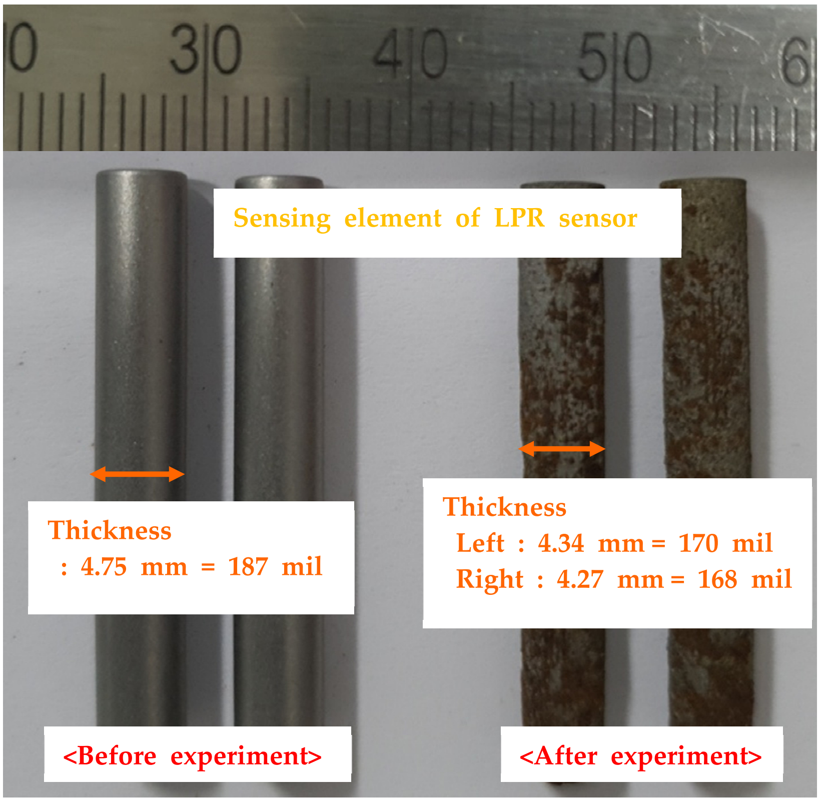

(b)

Figure S6. Photographs of (a) Sensing element of ER sensor and (b) Sensing element of LPR sensor after the test.

	Bottom	Mid	Top
3D analysis	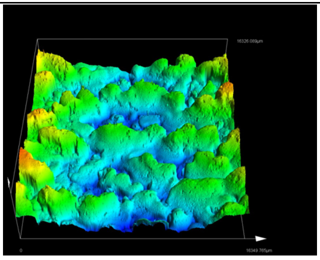	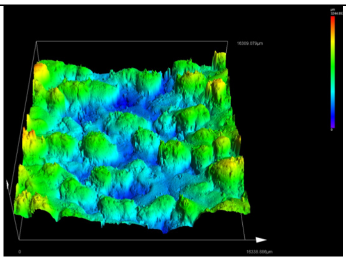	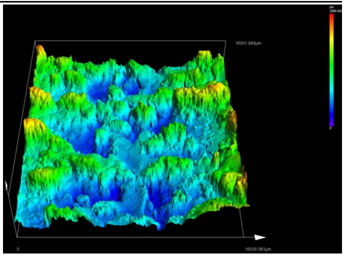
Surface roughness	408.902 μm	391.747 μm	357.945 μm
Height histogram	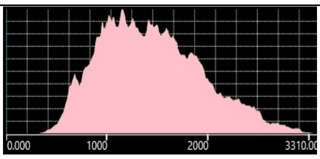	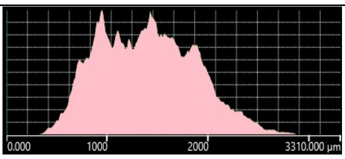	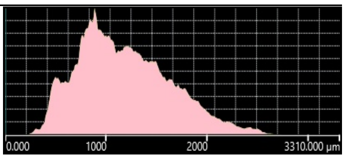

Figure S7. Surface analysis of the inner side of the test pipe using 3D laser confocal microscope.
